# Supplementary material for: Synchrony and symmetry-breaking in active flagellar coordination
Source: Philos Trans R Soc Lond B Biol Sci. 2019 Dec 30;375(1792):20190393. doi: 10.1098/rstb.2019.0393 (PMC7017343; doi:10.1098/rstb.2019.0393)
Supplement: Supplementary methods and figures [file rstb20190393supp1.pdf]

Supplementary Materials (SM)

1. Experimental Methods

The following lists all the multiflagellate species used for this study:

Biflagellates: *Chlamydomonas reinhardtii, Polytoma uvella*

Quadriflagellates: *Pyramimonas parkeae, Tetraselmis suecica, Tetraselmis subcordiformis, Carteria crucifera*

Octoflagellate: *Pyramimonas octopus*

Hexadecaflagellate: *Pyramimonas cyrtoptera**
(*Note: the hexadecaflagellate *Pyramimonas cyrtoptera* perished in culture in 2017, and has not been recoverable since. The species is also lost to the Collection Centre.)

Organisms were cultured as described previously [15]. In addition, the quadriflagellate species *Pyramimonas tychotreta* [42] (SCCAP K-0383, Scandinavian Culture Collection of Algae and Protozoa), was acquired specifically for this study.

Imaging and micromanipulation methods were as described in [15]. Briefly, free-swimming organisms were imaged in sealed square chambers (with glass coverslips above and below) with dimensions of approx. 1.5 cm x 1.5 cm, and a depth of approx. 50 μm. For the micropipette experiments, larger chambers were used to facilitate pipette access, these were also approx. 1.5 cm x 1.5 cm, but had a much larger depth of ~0.3 cm.

2. Selective activation

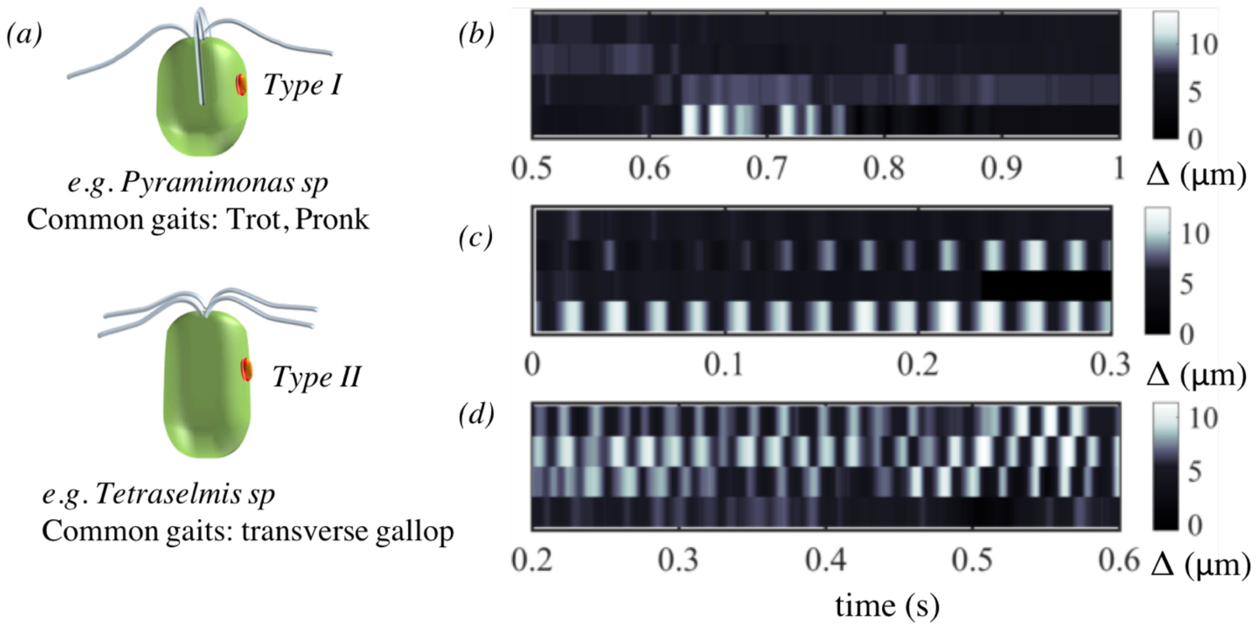

Figure S1: a) Here we summarise the two main flagellar configurations found in quadriflagellates and their respective forward swimming gaits. We provide further examples of selective gait activation in the quadriflagellate *Pyramimonas tychotreta* (type I), in which beating is restricted to one (b), two (c), or three (d), of a total possible four flagella. Data provided as supplementary movies SV1-3. An additional movie SV4 shows the canonical trot gait of *P. tychotreta*, which involves all four flagella.

3. Another example of gait mechanosensitivity

In *P. parkeae,* mechanical perturbations can also induce a shock response. (See also section 3 of main text.)

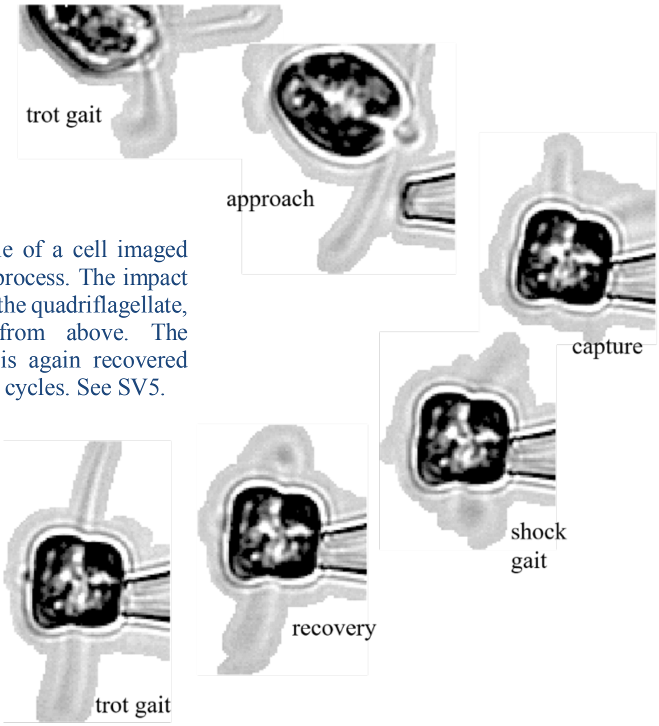

Figure S2: Another example of a cell imaged during the microaspiration process. The impact induces a shock response in the quadriflagellate, which here is viewed from above. The characteristic trotting gait is again recovered after a small number of beat cycles. See SV5.

4. Timescales for gait switching

To determine average timescales for gait-switching. Trajectory segments corresponding to gait-transitions were extracted and aligned for two different quadriflagellate species.

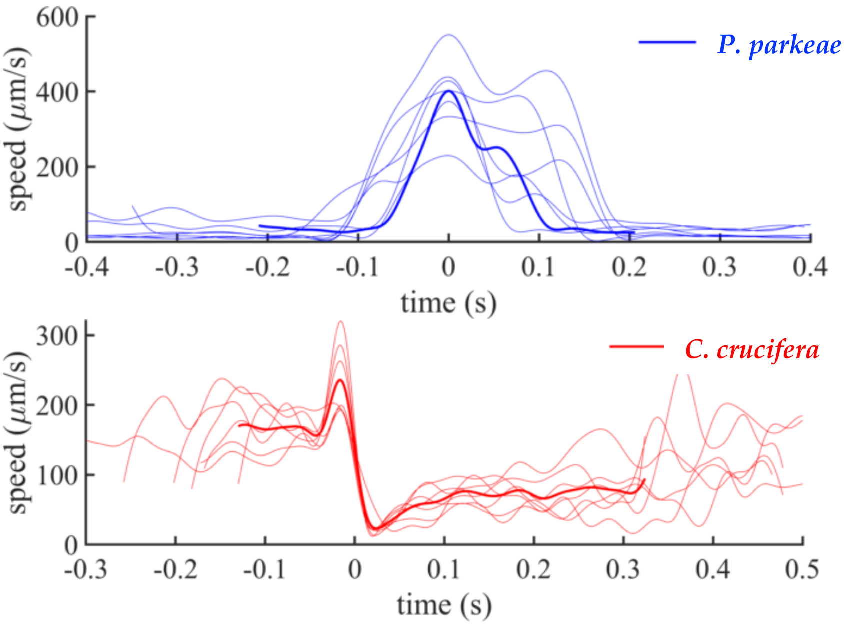

Figure S3: a) For *P. parkeae,* which shows intermittent swimming, bursting events in the swimming speed corresponds to beat activation in at least one flagellum.

b) Trajectory reorientations in *C. crucifera* occurs on a similar timescale. The timescale for the shock response itself (negative slope) is surprisingly conserved across the population.

5. Symmetry breaking in *P. octopus*.

As discussed in the main text, the rotary breaststroke of *P. ocotopus* is noisy – and cells often deviate from the canonical phase correlation pattern during free-swimming. Here, we present an example of when a more unusual pattern of synchrony is observed, where multiple adjacent flagella beat synchronously (Figure S4).

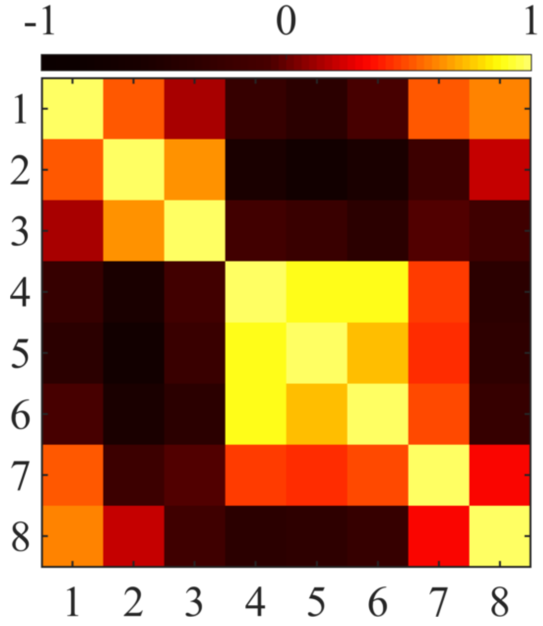

Figure S4: Phase correlation matrix for the cell in SV6.

The following multimedia files (SV1-7) accompany the SM:

1. Quadriflagellate gait with 1/4 flagella active (Figure 1b *Pyramimonas tychotreta*)
2. Quadriflagellate gait with 2/4 active (Figure 1c *Pyramimonas tychotreta*)
3. Quadriflagellate gait with 3/4 active (Figure 1d *Pyramimonas tychotreta*)
4. Quadriflagellate gait with 4/4 active (*Pyramimonas tychotreta*)
5. Quadriflagellate being caught by micropipette (Figure 2 *Pyramimonas parkeae,* viewed from above)
6. Octoflagellate "phase slip"
7. Octoflagellate search gait – extended flagellum (another example).
